# Freedom of Atrial Fibrillation Predictions After Pulmonary Vein Isolation: A Review of Current Evidence

**DOI:** 10.31083/RCM36588

**Published:** 2025-05-21

**Authors:** Ibrahim Antoun, Ahmed Abdelrazik, Mahmoud Eldesouky, Ahmed I. Kotb, Zakkariya Vali, Abdulmalik Koya, Edward Y. M. Lau, Ivelin Koev, Riyaz Somani, G. André Ng

**Affiliations:** ^1^Department of Cardiology, University Hospitals of Leicester NHS Trust, Glenfield Hospital, LE3 9QP Leicester, UK; ^2^Department of Cardiovascular Sciences, Clinical Science Wing, University of Leicester, Glenfield Hospital, LE3 9QP Leicester, UK; ^3^Department of Research, National Institute for Health Research Leicester Research Biomedical Centre, LE3 9QP Leicester, UK

**Keywords:** atrial fibrillation, direct current cardioversion, outcomes, electrocardiogram

## Abstract

Atrial fibrillation (AF), the most common sustained cardiac arrhythmia, poses significant challenges due to high morbidity, mortality, and healthcare costs. Pulmonary vein isolation (PVI) is a cornerstone treatment that disrupts arrhythmogenic pathways through electrically isolating pulmonary veins. However, recurrence rates remain substantial, driven by complex demographic, biochemical, imaging, and electrocardiographic factors reflecting underlying pathophysiologies. Advancements in PVI techniques, including pulsed-field ablation and electroanatomic mapping, have improved procedural success. Antiarrhythmic drugs (AADs) enhance outcomes by stabilising atrial activity and reducing early recurrence, although the long-term benefits of these drugs are debated. Nonetheless, integrating these predictors into patient selection, procedural strategies, and post-ablation management enables personalised interventions. This review uniquely integrates demographic, biochemical, imaging, electrocardiographic, and procedural predictors into a multidimensional framework for comprehensive risk stratification of PVI outcomes. We critically evaluate emerging procedural techniques, notably pulsed-field ablation (PFA), emphasising the clinical applicability of these procedures. Key biochemical markers (e.g., N-terminal pro-brain natriuretic peptide (NT-pro-BNP), C-reactive protein (CRP), interleukin-6 (IL-6)) and imaging findings (e.g., left atrial fibrosis, epicardial fat) reflecting atrial pathophysiology are discussed in detail. Furthermore, readily accessible electrocardiographic parameters such as prolonged P wave duration and dispersion are emphasised as practical tools for patient risk assessment. This multidimensional approach holds promise for reducing AF recurrence and improving long-term outcomes in PVI, advancing patient-centered care in AF management.

## 1. Introduction

Atrial fibrillation (AF) is the most prevalent sustained cardiac arrhythmia, affecting millions of individuals worldwide and significantly contributing to morbidity, mortality, and healthcare costs [1]. The prevalence is increasing, especially in the developing world, where management is challenging. Pulmonary vein isolation (PVI) has emerged as a cornerstone in the interventional treatment of AF, aiming to electrically isolate the pulmonary veins (PVs) from the left atrium (LA), thereby disrupting the initiation and maintenance of arrhythmia [2]. Despite advancements in ablation techniques, reducing the burden of AF remains challenging, with recurrence rates differing based on patient characteristics and procedural factors. The success of PVI is influenced by a complex interplay of demographic, biochemical, imaging, and electrocardiographic elements that reflect the underlying pathophysiological mechanisms driving AF. These mechanisms include atrial remodelling, fibrosis, inflammation, and conduction abnormalities, all of which can affect the effectiveness of PVI and predispose patients to recurrence [3]. Understanding these predictors is vital for refining patient selection, enhancing procedural outcomes, and optimising long-term management strategies. The literature has studied PVI outcome predictors extensively. However, no robust scoring system has been implicated, increasing the need for large-scale studies. It is essential to distinguish between procedural failure (incomplete PVI or acute reconnection) and clinical outcomes such as AF recurrence and AF burden reduction. AF burden, defined as the proportion of time an individual spends in AF, has emerged as a critical clinical endpoint, strongly correlating with patient outcomes, including stroke, heart failure, quality of life, and mortality. Recent guidelines and studies emphasise the significance of reducing the AF burden rather than solely achieving absolute freedom from AF recurrence. Consequently, this review incorporates evidence related to demographic, biochemical, imaging, electrocardiographic, and procedural predictors in terms of recurrence and, importantly, in the context of their impact on overall AF burden [4, 5]. Despite significant advancements in PVI, AF recurrence rates remain up to 30% [6], reflecting substantial limitations in current procedural methods. These high recurrence rates contribute to repeated procedures, increased healthcare costs, and ongoing patient morbidity [7]. Therefore, the precise prediction of procedural outcomes, including recurrence and AF burden reduction, remains critical yet challenging in clinical practice. Accurately identifying patients most likely to benefit from PVI would significantly optimise clinical outcomes, resource utilisation, and patient-centred care. Incorporating the latest clinical guidelines emphasises the urgency of developing robust predictive frameworks, supporting individualised management strategies and improving overall success rates. Thus, this review synthesises available evidence to identify and integrate key demographic, biochemical, imaging, electrocardiographic, and procedural predictors, aiming to address these critical gaps and advance clinical decision-making. This multidimensional approach supports enhanced patient stratification and personalised management strategies and aligns with contemporary clinical objectives in AF treatment. By integrating these findings, we seek to improve clinical decision-making, highlight areas for further research, and support the development of individualised treatment approaches to improve outcomes for patients undergoing PVI.

## 2. Demographics

A summary of studies on demographics related to the lack of AF burden reduction is presented in Table 1 (Ref. [8, 9, 10, 11, 12, 13, 14, 15, 16, 17, 18, 19, 20, 21, 22, 23, 24, 25]). Many patients undergoing PVI have potentially modifiable risk factors, highlighting the need to address these factors to minimise AF recurrence after ablations [26]. Various factors linked to suboptimal outcomes have been identified across different populations, offering insights into patient selection and individualised management strategies. Key demographic predictors of lack of AF burden reduction include age and sex. Increased age has been shown to diminish quality of life post-PVI in up to 20% of elderly patients, as reported by Vermeersch *et al*. [8]. Furthermore, the female sex appears to be a strong predictor of lack of AF burden reduction, potentially exacerbated by anatomical and hormonal factors [9, 10].

**Table 1.** **The role of demographics in predicting reduced AF burden after pulmonary vein isolation**.

Study	Demographics associated with lack of atrial fibrillation burden reduction
Themistoclakis *et al*., 2008 [11], Bahnson *et al*., 2022 [12]	Non-PAF, hypertension, AF duration ↑
Tuan *et al*., 2010 [13], Vermeersch *et al*., 2021 [8]	Age ↑, 20% of elderly patients had meaningful ↓ QoL over one year
Creta *et al*., 2020 [14]	Diabetes mellites
Ng *et al*., 2011 [15]	Obstructive sleep apnoea
D’Ascenzo *et al*., 2013 [16]	Recurrence within 30 days, valvular AF
Jacobs *et al*., 2015 [17]	CHA_2_DS_2_-Vasc ↑
Li *et al*., 2014 [18]	Chronic kidney disease
Qiao *et al*., 2015 [19]	Alcohol intake ↑
Sultan *et al*., 2017 [20]	In hospital recurrence, females, non-PAF
Pallisgaard *et al*., 2018 [10]	Female sex, hypertension, AF duration >2 years, Cardioversion <1 year of ablation
Winkle *et al*., 2017 [21], Pranata *et al*., 2021 [22]	Obesity, Body mass index ≥35 kg/m^2^
Kuck *et al*., 2018 [9], Li *et al*., 2020 [23]	Female sex, Height in females
Kim *et al*., 2020 [24]	Anaemia before ablation
Chew *et al*., 2020 [25]	Diagnosis to ablation time ↑

PAF, paroxysmal atrial fibrillation; AF, atrial fibrillation; QoL, quality of life. ↑: increase. ↓: decrease.

Furthermore, a recent meta-analysis involving 6819 patients suggested that females have a lower PVI success rate [27]. Additionally, females had more comorbidities and were more symptomatic of AF [28, 29]. The elderly population exhibited a higher probability of lack of AF burden reduction without prognostic benefits [8]. Certain comorbidities serve as critical predictors. Hypertension, diabetes mellitus, and obesity are consistently linked to PVI failure, likely due to their effects on atrial remodelling and fibrosis.

Furthermore, conditions such as obstructive sleep apnoea (OSA), chronic kidney disease, and anaemia are associated with poorer outcomes [15, 18, 24]. Temporal factors and disease progression also play a crucial role. Prolonged AF duration, shorter time intervals between diagnosis and ablation, and cardioversion within one year of ablation predict poor PVI outcomes. In-hospital and early AF recurrence within 30 days post-ablation are also significant predictors of lack of AF burden reduction [25]. Lastly, lifestyle factors, such as increased alcohol intake and a family history of AF, further highlight the multifactorial nature of PVI outcomes [19]. The CHA₂DS₂-VASc score and structural factors like valvular AF are additional independent predictors [17]. These studies highlight the significance of demographic and clinical characteristics in identifying patients at a higher risk of PVI failure. Customising pre-procedural assessments and optimising modifiable risk factors may improve procedural success and long-term outcomes. Another crucial factor affecting procedural success is the arrhythmic burden prior to the ablation. Increased frequencies of AF episodes before the procedure are associated with greater enhancements in arrhythmia-specific symptoms and health-related quality of life after ablation [30]. This correlation suggests that patients with more frequent AF episodes may experience a more substantial reduction in arrhythmic burden, thereby enhancing the perceived success of the procedure. However, there was no substantial correlation between AF burden and quality of life [5]. Future systematic reviews incorporating meta-analysis are recommended to quantify the impact of these demographic factors on AF recurrence and burden accurately, thus enabling more precise clinical risk stratification and targeted patient management.

## 3. Procedural Aspects

**Fig. 1.**
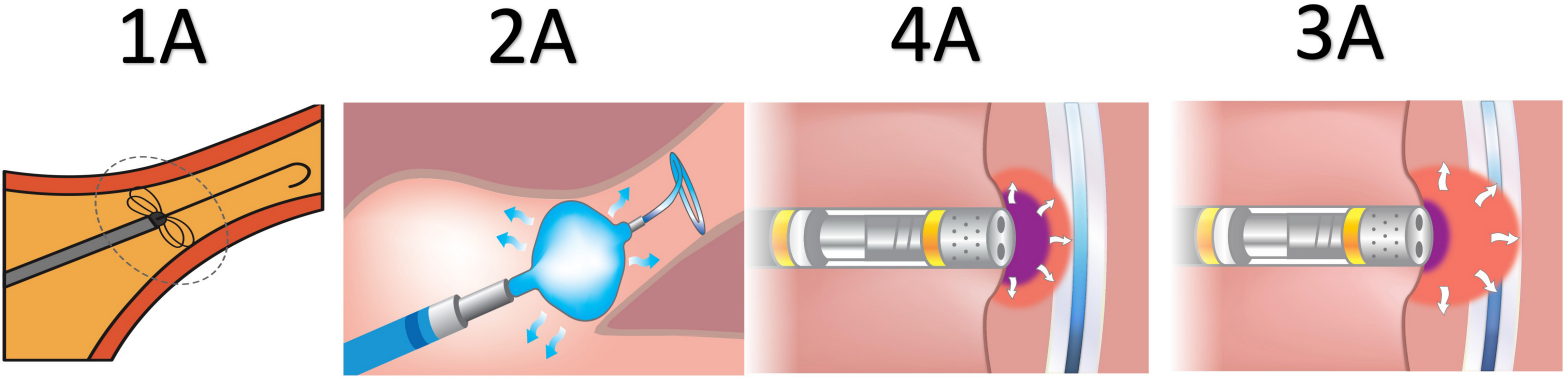
**Different modalities of pulmonary vein isolation for atrial fibrillation**. (1A) Pulsed-field ablation. (2A) Cryoballoon ablation. (3A) Very high-powered short-duration ablation. (4A) Radiofrequency ablation.

According to a recent meta-analysis, heart failure constitutes a large burden of AF treatment costs [17]. Recent evidence highlights the clinical significance of catheter ablation in patients with AF and concurrent heart failure. The CASTLE-AF trial demonstrated that PVI notably reduced mortality and hospitalisation due to worsening heart failure compared with medical therapy alone in symptomatic patients with reduced ejection fraction [31]. Similarly, the CASTLE-HTx trial extended these findings to patients with advanced, end-stage heart failure awaiting heart transplantation, revealing a significant reduction in mortality, urgent transplantation, or ventricular assist device implantation with catheter ablation [32]. Furthermore, Bergonti *et al*. [33] externally validated a straightforward four-parameter clinical scoring model (the Antwerp score) that effectively predicts left ventricular functional recovery after AF ablation, facilitating enhanced clinical decision-making in heart failure patients. These studies collectively reinforce PVI as a valuable treatment strategy in AF-related heart failure. Recent advancements in PVI have involved multiple catheters used over the years (Fig. 1). The literature has garnered considerable attention regarding anatomical predictors, particularly the correlation between pulmonary vein (PV) size and the likelihood of AF recurrence following ablation. Notably, larger right PVs have been identified as significant predictors of recurrence in patients with persistent AF [34]. These anatomical characteristics are critical as they inform the ablation strategy and help identify patients who may benefit from more aggressive treatment approaches. Procedural factors also play a crucial role in predicting PVI success. The temperature reached during cryoablation has been identified as a significant predictor; for instance, achieving a temperature of –40 °C within 30 seconds is linked to higher rates of initial PVI success [35]. These procedural metrics provide actionable insights that can be utilised during the ablation process to enhance outcomes. Physiological factors, particularly those related to the heart’s electrical activity, are also significant predictors. The presence of dissociated PV activity after PVI has been linked to a greater likelihood of arrhythmia recurrence, indicating that monitoring this activity may be critical for predicting long-term success [36]. Furthermore, acute PV reconnection has been identified as a significant predictor of atrial fibrillation recurrence, highlighting the necessity for vigilant monitoring during and after the procedure [37]. The choice of catheter plays a crucial role in the success of radiofrequency ablation (RF). Irrigated-tip catheters enable improved temperature control and lesion formation, which are vital for achieving transmural lesions. Studies indicate that these catheters can create deeper lesions while minimising the risk of thermal injury [38]. Accurate mapping of the LA is vital for identifying arrhythmogenic foci. Electroanatomic mapping systems provide a three-dimensional view of the heart’s anatomy, facilitating precise catheter placement and lesion delivery. The integration of contact force sensing technology has also enhanced outcomes by ensuring proper contact between the catheter and myocardial tissue, which is crucial for effective lesion formation. The power and duration of energy delivery during ablation are essential to lesion formation. Research indicates that higher power settings and longer application times can result in more durable lesions, although they also elevate the risk of complications [39].

Localised sources of arrhythmia, particularly in persistent AF, necessitate more extensive ablation strategies, which can complicate the procedure and affect outcomes [40]. A novel advancement in this field is pulsed-field ablation (PFA), which has emerged as a promising technique for PVI (Fig. 1). Unlike traditional thermal-based methods, PFA uses high-voltage, short-duration electrical pulses to ablate myocardial tissue while sparing surrounding structures selectively. This non-thermal approach reduces the risk of complications such as oesophageal injury and phrenic nerve damage. Studies have demonstrated that PFA achieves durable lesions with shorter procedure times and improved outcomes, making it a compelling alternative to conventional techniques [41, 42]. The specificity of PFA for cardiac tissue and its safety profile position it as a transformative technology in the realm of AF ablation [43]. Recent meta-analyses compared various ablation modalities in terms of safety and outcomes. For example, Tokavanich *et al*. [44], demonstrated that very-high powered short duration (vHPSD) ablation did not offer higher efficacy than high power short duration ablation (HPSD) and conventional RF. vHPSD ablation outcomes were comparable with techniques in a meta-analysis of 9721 patients [44]. Another meta-analysis of 3805 patients demonstrated that compared to cryoballoon ablation (cryo), PFA offered shorter procedure times and lower arrhythmia recurrence with a decreased periprocedural complications risks [45]. A third meta-analysis of 109 studies has shown that cryo, PFA and laser balloon have demonstrated shorter procedure times with superior efficacy and comparable safety compared to conventional RF [46].

## 4. Biochemical Factors

Biochemical markers associated with AF recurrence are summarised in Table 2 (Ref. [47, 48, 49, 50, 51, 52, 53, 54, 55, 56, 57, 58, 59, 60, 61]), including those linked to inflammation, fibrosis, and cardiac remodelling. These markers provide valuable insights into the mechanisms underlying lack of AF burden reduction after PVI. Research has identified inflammation, fibrosis, atrial remodelling, and genetic predisposition markers as significant predictors of poor outcomes. Elevated levels of inflammatory markers such as C-reactive protein (CRP), interleukin-6 (IL-6) promote atrial electrical and structural remodelling by activating fibroblasts and facilitating extracellular matrix deposition, thereby creating a substrate conducive to reentry circuits [47]. Also, tissue inhibitor of metalloproteinase-2 (TIMP-2) have been associated with higher recurrence rates following PVI, as they are directly involved in collagen synthesis and atrial fibrosis, leading to impaired conduction and heightened arrhythmogenic potential following ablation [62]. Likewise, fibrosis markers, including transforming growth factor beta 1 (TGF-β1), soluble suppression of tumorigenicity 2 protein (ST2), and visfatin, have been related to adverse outcomes, highlighting the significance of atrial fibrosis in procedural success [48, 49, 50]. Cardiac stress biomarkers like N-terminal pro-brain natriuretic peptide (NT-pro-BNP), brain natriuretic peptide (BNP), and atrial natriuretic peptide (ANP) were consistently elevated in patients with poor outcomes, reflecting elevated atrial pressure and stretch, leading to progressive atrial enlargement and remodeling, thereby heightening susceptibility to AF recurrence [51]. Genetic factors also play a crucial role, with the 4q25 mutation and altered paired-like homeodomain transcription factor 2 ( *PITX2*) messenger RNA (mRNA) expression levels being associated with higher recurrence risks [52].

**Table 2.** **Biochemical predictors of reduced AF burden after pulmonary vein isolation**.

Study	Biochemical markers associated with lack of atrial fibrillation burden reduction
Nakazawa *et al*., 2009 [53]	Endothelin-1 ↑ (vasoconstrictor associated with left atrial remodelling)
Tang *et al*., 2010 [54]	High normal thyroid status
Husser *et al*., 2010 [52]	4q25 mutation
Wang *et al*., 2019 [55]	Myocardial collagen turnover marker (Metalloproteinase-2)
Wu *et al*., 2013 [47], Jiang *et al*., 2017 [56], Zhang *et al*., 2016 [51]	N-terminal pro-brain natriuretic peptide ↑, brain natriuretic peptide ↑, atrial natriuretic peptide ↑, C-reactive protein ↑, interleukin-6 ↑, and tissue inhibitor of metal loproteinase-2 ↑, low density lipoprotein ↑
Canpolat *et al*., 2015 [57]	Monocyte to high-density lipoprotein ratio ↑
Tian *et al*., 2017 [49]	Transforming growth factor B1 ↑ (fibrosis marker)
Okar *et al*., 2018 [48]	Soluble ST2 ↑ (fibrotic marker)
Platek *et al*., 2020 [58]	Visfatin ↑ (adipokine made by visceral fat which played a role in inflammation and fibrosis)
Shang *et al*., 2020 [59]	low density lipoprotein ↓, total cholesterol ↓ in females
Reyat *et al*., 2020 [60]	mRNA plasma *PITX2* ↑, mRNA left atrial *PITX2* ↓ (cardiac transcription factor)
Suehiro *et al*., 2021 [50]	Intermediate monocytes ↑ (profibrotic marker)
Wang *et al*., 2021 [61]	Carbohydrate antigen-125 ↑

mRNA, messenger ribonucleic acid; *PITX2*, paired-like homeodomain transcription factor 2. ↑: increase. ↓: decrease.

Furthermore, abnormal myocardial collagen turnover, indicated by elevated metalloproteinase-2, has been associated with atrial remodelling and procedural failure [55]. Metabolic markers have also been linked to poor outcomes, such as elevated low-density lipoprotein (LDL) and a high monocyte-to-high-density lipoprotein (HDL) ratio. This further exacerbates inflammation-driven remodelling and endothelial dysfunction, ultimately reducing procedural efficacy [57]. Paradoxically, lower LDL and total cholesterol levels in females predicted failure, suggesting sex-specific influences [59]. Other markers, such as endothelin-1, carbohydrate antigen-125, and intermediate monocytes, further emphasise the multifaceted nature of PVI outcomes. These findings highlight the significance of biochemical profiling in predicting PVI success, facilitating tailored approaches to enhance long-term outcomes in AF management.

## 5. Cardiovascular Imaging

Imaging modalities are essential for identifying structural and functional abnormalities linked to lack of AF burden reduction. A thorough evaluation of these imaging findings unveils significant predictors that indicate underlying atrial remodelling, fibrosis, inflammation, and haemodynamic stress, all of which contribute to procedural lack of AF burden reduction and recurrence of AF (Table 3, Ref. [16, 63, 64, 65, 66, 67, 68, 69, 70, 71, 72, 73, 74]). Structural remodelling of the LA is widely recognised as a key factor influencing PVI outcomes. An enlarged LA, demonstrated by increased size, diameter, and volume, consistently predicts lack of AF burden reduction [75]. These findings emphasise the connection between LA dilatation and advanced atrial remodelling, which may lead to the persistence of AF even after successful ablation. AF is an underappreciated reversible cause of left ventricle systolic dysfunction in this population despite adequate rate control. According to the CAMERA-MRI study, restoring sinus rhythm with PVI significantly improves ventricular function in patients without ventricular fibrosis on cardiac magnetic resonance (CMR), stressing the role of PVI in these patients [76]. This supports the role of imaging in improving PVI outcomes.

**Table 3.** **Correlation between imaging studies and reduced AF burden after pulmonary vein isolation**.

Study	Imaging associated with lack of atrial fibrillation burden reduction
Bertaglia *et al*., 2005 [63]	Structural heart disease
Schneider *et al*., 2008 [64]	LA strain and strain rate ↓
Wong *et al*., 2011 [65]	Presence of pericardial fat
D’Ascenzo *et al*., 2013 [16]	LA diameter ↑
Shin *et al*. 2008 [66]	LA volume ↑
Chelu *et al*., 2018 [67], Kheirkhahan *et al*., 2020 [68], Ghafouri *et al*., 2021 [69]	Fibrosis detected by CMR
Sepehri Shamloo *et al*., 2019 [70], Kawasaki *et al*., 2020 [71]	Epicardial fat tissue volume and thickness ↑
Mouselimis *et al*., 2020 [72]	LA strain ↑
Weyand *et al*., 2025 [73]	Tricuspid regurgitation
Shchetynska-Marinova *et al*., 2022 [74]	LA size ↑, atrial stiffness ↑

LA, left atrium; CMR, cardiac magnetic resonance. ↑: increase. ↓: decrease.

Functional impairment of the LA, especially diminished strain and strain rate, has also emerged as a significant predictor of adverse outcomes. These markers indicate decreased compliance and contractility of the atrial myocardium, further hindering the success of PVI. Furthermore, the right atrial area has been suggested as a new predictive variable, with studies showing that an enlarged right atrial area correlates with an increased risk of AF recurrence following PVI [77]. Atrial fibrosis, identified through CMR, is another crucial predictor of lack of AF burden reduction [78]. Fibrotic changes in the atrium lead to electrical and structural remodelling, potentially promoting AF recurrence by facilitating re-entry circuits and ectopic activity. Imaging studies have affirmed that greater degrees of fibrosis are strongly associated with lower ablation success rates. Likewise, impaired scar formation, which indicates an inadequate atrial tissue response post-ablation, has been linked to recurrence, highlighting the importance of atrial tissue integrity in achieving lasting results. Visualised through imaging, inflammatory markers enhance the understanding of PVI outcomes. Increased epicardial fat volume and thickness have been demonstrated to correlate with recurrence, as these tissues release pro-inflammatory cytokines that can exacerbate atrial remodelling [79]. The presence of pericardial fat has similarly been associated with poorer outcomes, likely due to its role in modulating the local inflammatory environment of the atria. Advanced imaging of these fat deposits provides valuable insights into systemic and local inflammation contributing to the lack of AF burden reduction. Hemodynamic factors identified through imaging include tricuspid regurgitation and increased atrial stiffness, which indicate elevated atrial pressure and impaired ventricular-atrial coupling [73, 74]. These hemodynamic stressors may sustain atrial remodelling and fibrosis, ultimately diminishing the effectiveness of PVI. Imaging can detect subtle changes in cardiac function, such as impaired LA strain, which may precede overt structural abnormalities and offer an early warning of poor outcomes. These findings underline the necessity of advanced imaging techniques in pre-procedural evaluation and risk stratification for PVI. By pinpointing structural, functional, and inflammatory predictors of lack of AF burden reduction, imaging allows clinicians to customise treatment strategies to individual patient profiles. For instance, patients with significant LA enlargement or extensive fibrosis may gain from adjunctive therapies, such as anti-arrhythmic medications or hybrid surgical catheter ablation approaches. Additionally, imaging-guided interventions could address modifiable risk factors, such as controlling inflammation or optimising atrial pressures, to enhance procedural outcomes. Emerging imaging technologies, particularly artificial intelligence (AI)-assisted imaging and advanced computational techniques, represent promising frontiers in predicting AF recurrence following PVI [80]. AI algorithms used in CMR, computed tomography (CT), and echocardiography have shown superior capabilities in detecting subtle patterns of fibrosis, structural remodelling, and functional impairment that are not readily apparent with traditional methods [81]. These automated analytical approaches facilitate more precise and reproducible quantification of atrial remodelling, epicardial adipose tissue, and fibrosis burden, thereby significantly enhancing patient-specific risk assessment. Incorporating AI-assisted imaging into routine clinical evaluation may allow for earlier detection of high-risk patients, enabling tailored procedural strategies, improved patient selection, and potentially better long-term outcomes following AF catheter ablation.

In summary, imaging predictors of lack of AF burden reduction after PVI provide a comprehensive framework for understanding AF’s structural and functional foundations. By incorporating these findings into clinical practice, physicians can more effectively identify and refine procedural techniques and high-risk patients and develop personalised management strategies to reduce long-term AF burden. Advanced technologies such as intracardiac echocardiography have been shown to improve procedural outcomes by providing real-time imaging [82].

## 6. Antiarrhythmic Drugs Use

Anti-arrhythmic drugs (AADs) have emerged as valuable adjunctive therapies to enhance the success rates of PVI by addressing challenges in both the periprocedural and long-term management phases. AADs can improve procedural outcomes by stabilising atrial electrical activity during PVI, facilitating the identification and ablation of arrhythmogenic foci [83]. For instance, class Ic agents (flecainide, propafenone) can suppress ectopic atrial activity, thereby improving mapping accuracy, while class III agents (specifically amiodarone) prolong refractoriness, reducing the risk of acute recurrence [51]. These properties are particularly beneficial in patients with persistent AF, where atrial substrate modification may be more complex.

In the post-ablation period, AADs play a critical role in suppressing early recurrences of AF (ERAF), often due to transient inflammation or incomplete lesion formation. Studies suggest that short-term AAD therapy following PVI can reduce the incidence of ERAF, potentially improving long-term rhythm outcomes. Clinical trials have yielded mixed results regarding the routine use of AADs post-PVI, underscoring the importance of individualised treatment approaches. Although short-term use of AADs after AF ablation decreases ERAF, early use does not prevent recurrence after 6 months [84]. ERAFs occurring on or off AADs during the initial 6-week blanking period strongly influence long-term AF recurrence. For example, the AMIO-CAT trial demonstrated that short-term amiodarone therapy reduced ERAF but did not significantly impact long-term success rates [85]. Compared to amiodarone, sotalol and propafenone were associated with an increased risk of ERAF [86]. Although there was no significant difference in ERAF between dronedarone and amiodarone, dronedarone was suggested as the preferred option due to its lower frequency of side effects [87]. Additionally, the advent of hybrid strategies, combining PVI with AAD therapy tailored to the patient’s AF subtype and comorbidities, has shown promise in improving outcomes. Another study indicated that the rates of recurrences, cardiovascular events, and mortality did not differ between patients discharged with or without AAD following AF PVI. However, AAD therapy should be carefully considered in cases of paroxysmal AF, which has been associated with a higher rate of redo ablation and decreased treatment satisfaction [88].

**Fig. 2.**
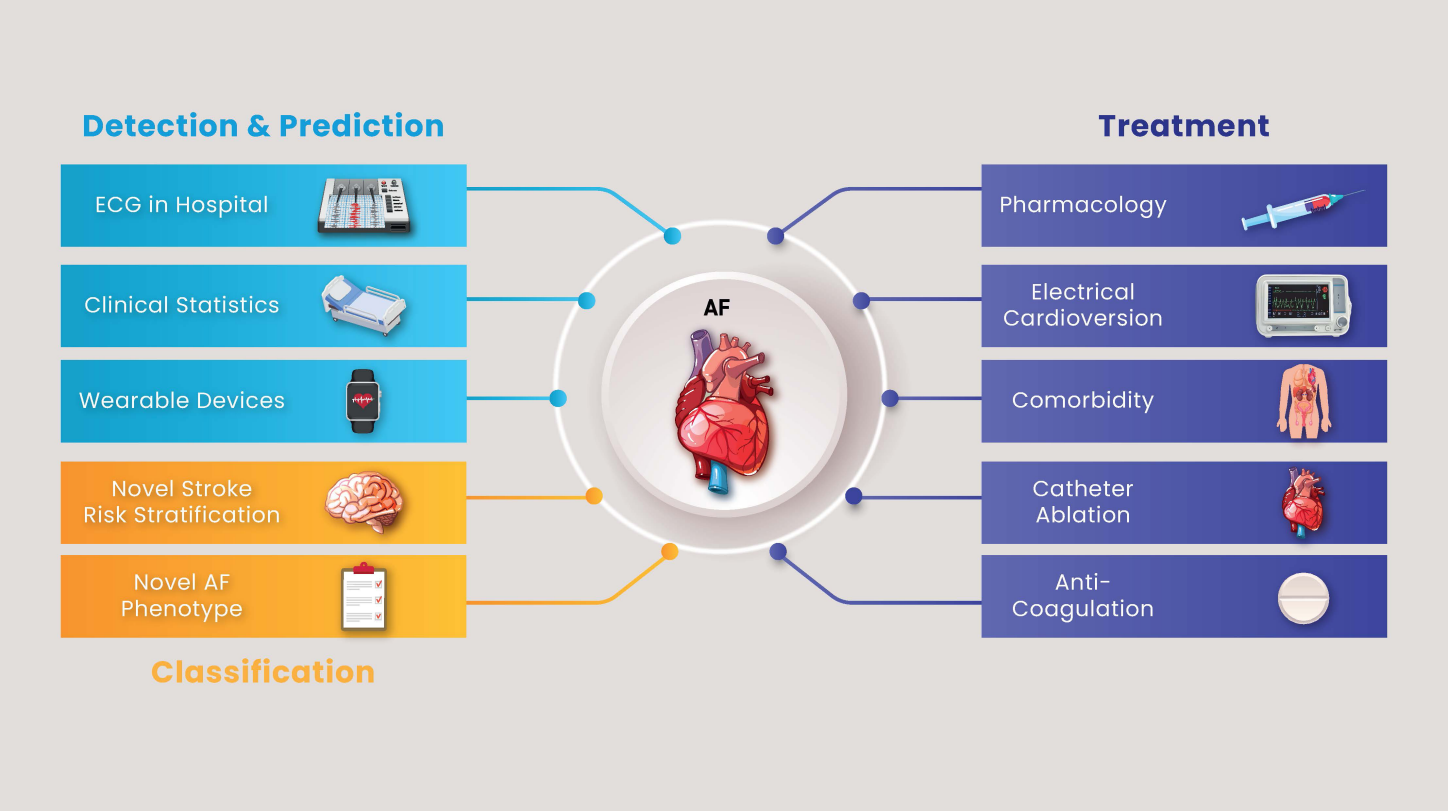
**Stepwise approach for diagnosing and treating atrial fibrillation**. AF, atrial fibrillation; ECG, electrocardiogram.

Despite their benefits, using AADs in the context of PVI is not without limitations. Adverse effects, proarrhythmia, and the potential for masking incomplete ablation underscore the need for careful patient selection and monitoring. It is important to acknowledge discrepancies in the literature regarding the long-term efficacy of AADs following PVI. While several studies have demonstrated that short-term AAD therapy effectively reduces early recurrences of AF, such as the EAST-AF trial [89], the long-term benefits remain contentious. Trials such as AMIO-CAT found temporary suppression of AF without sustained long-term rhythm control benefits. In contrast, other studies suggest potential advantages in selected patient populations or specific AF subtypes [83]. These inconsistencies may arise from variations in patient characteristics, duration of drug therapy, ablation techniques, and study methodologies. Clarifying these discrepancies through future prospective, randomised controlled trials is necessary to determine the optimal role and duration of AAD therapy after PVI, ultimately informing tailored, patient-specific treatment strategies. The process of personalised stepwise approach for atrial fibrillation is demonstrated in Fig. 2.

Future research should focus on identifying biomarkers and patient-specific factors that predict responsiveness to AADs in the peri- and post-ablation phases and exploring novel agents that may synergise with ablation strategies. Therefore, antiarrhythmics are integral to optimising the success of PVI for AF, particularly in high-risk patients. AADs complement ablation strategies by enhancing procedural precision, reducing ERAF, and supporting long-term rhythm stability. However, their application requires a nuanced approach, balancing potential benefits with risks and tailoring therapy to individual patient profiles.

## 7. Electrocardiogram Markers

Electrocardiogram (ECG) parameters have been extensively researched to predict PVI lack of AF burden reduction (Table 4, Ref. [62, 84, 85, 90, 91, 92, 93, 94, 95, 96, 97, 98, 99, 100, 101, 102, 103]). Prolonged P-wave duration (PWD), P-wave dispersion (PWDisp), and abnormal P-wave terminal force in lead V1 (PTFV1) have consistently emerged as indicators of atrial conduction abnormalities, which may contribute to higher recurrence rates following PVI. Prolonged PWD, pre- and post-procedure, has been the most examined parameter. Increased PWD, particularly above certain thresholds (e.g., >120 ms, >140 ms, or >150 ms) [98, 99, 100], has been linked to a greater risk of AF recurrence.

**Table 4.** **Correlation between P-wave parameters and reduced AF burden after pulmonary vein isolation**.

Author and year	n	Time	Recurrence	Cut-off
Jiang *et al*., 2006 [62]	108	Post	↑ PWDisp	
Ogawa *et al*., 2007 [90]	27	Post	↑ PWD	
Okumura *et al*., 2007 [91]	51	Pre	↑ PWD	>150 ms
Van Beeumen *et al*., 2010 [92]	39	Post	↑ PWD	≤5 ms change
Caldwell *et al*., 2013 [93]	100	Pre	↑ PWD	>140 ms
Salah *et al*., 2013 [94]	198	Post	↑ PWDisp ↓ PTFV1	>40 ms
			↑ PWD	≤–0.04 mV⋅ms
				PWD >120 ms
Blanche *et al*., 2013 [85]	102	Post	↑ PWD	PWD >140 ms
Mugnai *et al*., 2016 [84]	426	Post	↑ PWDisp	
			↑ PWD	
Hu *et al*., 2016 [95]	171	Post	↑ PWD	
Wu *et al*., 2016 [96]	204	Post	↑ PWD	
Kanzaki *et al*., 2016 [97]	76	Post	↑ PTFV1	>9.3 mm⋅s
Jadidi *et al*., 2018 [98]	72	Pre	↑ PWD	>150 ms
Yanagisawa *et al*., 2020 [99]	115	Post	↓ Then ↑ PWD	
Auricchio *et al*., 2021 [100]	282	Post	↓ PWD	<110 ms
Supanekar *et al*., 2022 [101]	160	Post	PR interval↑ and PWD↓	
Ohguchi *et al*., 2022 [102]	84	Post	↑ PWD	≥120 ms
Miao *et al*., 2022 [103]	273	Post	↑ PWD	

PWD, P wave duration; PTFV1, P-wave terminal force in V1; PWDisp, P-wave dispersion. ↑: increase, ↓: decrease.

Studies have shown these thresholds correlate with delayed conduction across the atria and structural remodelling. Additionally, PWD changes after PVI (e.g., ≤5 ms change or a reduction below 110 ms) may reflect procedural effectiveness, with insufficient reduction linked to recurrence.

PWDisp, representing the variability in PWD across different leads, has also been identified as a predictor of PVI lack of AF burden reduction. Elevated PWDisp, both pre-and post-procedure, indicates heterogeneous atrial conduction and susceptibility to reentry circuits, contributing to AF recurrence. Abnormal PTFV1 has been another important marker, particularly when combined with prolonged PWD. An increased PTFV1 (e.g., >9.3 mm⋅s) or a reduction in PTFV1 below specific cut-offs (≤–0.04 mV⋅ms) has been associated with procedural failure, reflecting atrial conduction delay and remodelling localised to the LA [94, 97]. The evidence underscores the utility of ECG parameters in predicting PVI outcomes. These readily available and non-invasive markers offer a cost-effective approach to identifying patients at higher risk of recurrence. Future research should integrate these parameters with imaging and biochemical predictors for a comprehensive risk stratification model in atrial fibrillation management.

## 8. Conclusion

PVI is an essential tool in AF management, providing symptomatic relief and rhythm control. However, the challenge of recurrence highlights the need for a greater understanding of the factors contributing to the lack of reduction in AF burden. This review emphasises that demographic, biochemical, imaging, and electrocardiographic predictors are crucial in identifying patients at risk of suboptimal outcomes. Demographic factors such as age, sex, comorbidities, and the duration of atrial fibrillation provide essential context for patient stratification. Concurrently, biochemical markers reflect underlying inflammation, fibrosis, and atrial remodelling processes that influence procedural success. Imaging studies offer insights into structural and functional atrial abnormalities, with parameters such as LA size, fibrosis, and epicardial fat volume emerging as strong predictors of recurrence. Electrocardiographic features further enhance the capacity to predict PVI outcomes. These findings highlight the importance of a multidimensional approach to patient evaluation, combining clinical, biochemical, imaging, and electrocardiographic data for comprehensive risk stratification.

This review has several limitations that warrant acknowledgment. Firstly, the lack of ethnic and regional comparisons may limit the generalisability of findings, as variations in genetic, lifestyle, and healthcare delivery factors could significantly influence PVI outcomes. Future studies addressing ethnic diversity and regional variations are necessary for more inclusive and globally applicable conclusions. Secondly, while emerging ablation techniques such as PFA show promising results, uncertainties remain regarding the durability of lesion formation, long-term safety, and comparative effectiveness against already established ablation methods. Prospective randomised trials and real-world observational studies are required to clarify these uncertainties and confidently guide clinical practice. To facilitate the integration of demographic, biochemical, imaging, electrocardiographic, and procedural predictors into clinical practice, we recommend developing and validating comprehensive risk prediction tools or scoring systems. Such prediction models should leverage readily available clinical variables and new technologies, such as AI-assisted imaging modalities, to aid in stratifying patients according to their risk of AF recurrence and burden. Clinicians could utilise these tools to tailor ablation strategies, plan pre-procedurally, guide adjunctive therapies, and implement targeted follow-up care. Future research should prioritise multicentre validation studies and assess these prediction tools’ cost-effectiveness and clinical utility, promoting personalised AF management and improving patient outcomes.
